# Fish as predators and prey: DNA‐based assessment of their role in food webs

**DOI:** 10.1111/jfb.14400

**Published:** 2020-07-08

**Authors:** Michael Traugott, Bettina Thalinger, Corinna Wallinger, Daniela Sint

**Affiliations:** ^1^ Applied Animal Ecology, Department of Zoology University of Innsbruck Innsbruck Austria; ^2^ Centre for Biodiversity Genomics, University of Guelph Guelph Canada; ^3^ Institute of Interdisciplinary Mountain Research, Austrian Academy of Science Innsbruck Austria

**Keywords:** diet analysis, environmental DNA, food webs, metabarcoding, molecular gut content analysis, trophic networks

## Abstract

Fish are both consumers and prey, and as such part of a dynamic trophic network. Measuring how they are trophically linked, both directly and indirectly, to other species is vital to comprehend the mechanisms driving alterations in fish communities in space and time. Moreover, this knowledge also helps to understand how fish communities respond to environmental change and delivers important information for implementing management of fish stocks. DNA‐based methods have significantly widened our ability to assess trophic interactions in both marine and freshwater systems and they possess a range of advantages over other approaches in diet analysis. In this review we provide an overview of different DNA‐based methods that have been used to assess trophic interactions of fish as consumers and prey. We consider the practicalities and limitations, and emphasize critical aspects when analysing molecular derived trophic data. We exemplify how molecular techniques have been employed to unravel food web interactions involving fish as consumers and prey. In addition to the exciting opportunities DNA‐based approaches offer, we identify current challenges and future prospects for assessing fish food webs where DNA‐based approaches will play an important role.

## 1 INTRODUCTION

Fish are embedded in complex trophic networks, both as consumers of plants, invertebrates or other vertebrates and at the same time as prey and hosts for a wide range of species. Understanding these food web interactions is vital to comprehend the underpinning mechanisms driving alterations in fish communities in space and time (Baker *et al*., 2014). Compared to long‐term changes in community composition, such as species replacements or varying abundances of taxa, trophic shifts can happen rapidly, typically before transitions in the species assemblages are recognizable (Tylianakis *et al*., 2008). This means that the direct and indirect interactions between species and the species' functional roles do change without major changes in terms of community assemblages or before these occur. Therefore, quantifying these trophic links is essential to understand the effect of environmental perturbations, such as climate warming, on fish populations (Baker *et al*., 2014). To predict their future changes is furthermore crucial for the sustainable management of fish stocks (Baker *et al*., 2014). The importance of trophic links for the sustainable management of populations of both fish and piscivores has long been acknowledged. For example, large‐scale examinations of the diet of commercially relevant fish such as cod and herring have been implemented in long‐term monitoring programmes (*e.g*., Korneev *et al*., 2015) to provide dietary data for setting Barents Sea‐wide catching rates. Regarding piscivorous birds and mammals, their long‐standing perception as food competitors by humans (Duffy, 1995; Gosch *et al*., 2014) has led to an increase of shooting, culling, and repellent measures to reduce their populations in areas of conflict (Boudewijn and Dirksen, 1999; Bowen and Iverson, 2013). However, with many of them being endangered species, their management needs to be based on empirically generated dietary data to optimize and justify management interventions. For example, Grandquist *et al*. (2018) found no evidence of salmonids in the diet of harbour seals (*Phoca vitulina*) using a combination of DNA‐based and morphology‐based gut content analysis, calling for a reassessment of seal culling programmes that were implemented to reduce phocid predation on salmonids.

Key to any food web‐based approach is how trophic links are identified and quantified. The long‐term dietary patterns of both fish and piscivores have been examined by stable isotope and fatty acid analyses (Nielsen *et al*., 2018). Although these approaches can depict the feeding history of individuals, they are usually quite limited in the taxonomic resolution of the diet. More detailed diet information of fish has traditionally been obtained by examining the gut content for morphologically identifiable prey remains such as bones, scales, otoliths, exoskeletons and other hard part prey remains (Nielsen *et al*., 2018). The same is true for dietary studies on piscivores where, depending on species, the gut content of killed specimens (Boström *et al*., 2012; Labansen *et al*., 2007), regurgitated pellets (Dias *et al*., 2012; Gonzalez‐Solis *et al*., 1997; Thalinger *et al*., 2016) or faeces have been examined for identifiable hard parts (Jedrzejewska *et al*., 2001; Sinclair and Zeppelin, 2002). However, this type of diet analysis can be hampered by difficulties in identifying the prey remains. For example, small prey remains can be too digested to be identifiable while larger bits can be too distorted to achieve prey assignment on lower taxonomical levels as the food remains are too degraded for proper identification (Barrett *et al*., 2007; Bowen and Iverson, 2012). This is especially true for soft‐tissued prey such as jellyfish, small prey items and food which is easily digested often remains undetected by visual means (Berry *et al*., 2015; Zingel *et al*., 2012), leaving us with incomplete diet information. Moreover, the level at which this prey can be identified largely depends on the skills of taxonomists. Consequently, the trophic data generated may vary considerably between studies due to the differences in the capability of the persons involved. Finally, the possibility of automatizing morphological diet identification is limited by these conventional approaches, thus making diet analysis a time‐consuming and cumbersome process that hampers the routine analysis of the large data sets necessary to assess the dynamics in real‐world food webs and within routine monitoring programmes.

DNA‐based diet analysis constitutes a solution to most of these problems: DNA of the food of fish as well as DNA of their parasites or the one of fish being consumed by piscivores can be detected and identified at high specificity and sensitivity in different samples, including gut content, faeces or regurgitates. This enables the investigation of feeding interactions across trophic levels even within complex food webs at unprecedented resolution. Noteworthy for this special issue, aquatic ecologists have been at the forefront of DNA‐based diet analysis. Already in the 1990s the consumption of stone flounder *Kareius bicoloratus* by the sand shrimp *Crangon affinis* was examined by PCR‐based gut content analysis of the decapod (Asahida *et al*., 1997), while the field of DNA‐based diet analysis started to expand in the early 2000s (Agusti *et al*., 1999; Roslin and Majaneva, 2016; Zaidi *et al*., 1999).

Compared to nonmolecular approaches DNA‐based assessment of trophic interactions includes a range of advantages: (a) higher specificity and sensitivity at which food DNA can be detected and identified; (b) the ability to standardize the methodology; (c) the ability to verify the food and parasites detected *via* DNA sequences; and (d) the possibility of employing high‐throughput analyses. Due to the high sensitivity of the molecular methods, even a few molecules of food DNA can be detected (Thalinger *et al*., 2016), which allows a comprehensible assessment of what has been consumed compared to morphology‐based identification (Deagle *et al*., 2009). Moreover, DNA typically facilitates identifying prey at much lower taxonomic levels than hard part prey analysis (Berry *et al*., 2015), again leading to a more complete food spectrum of the species of interest. The application of DNA‐based methods for prey detection also allows for assessing trophic interactions in an unambiguous and verifiable way as the detected DNA is or can be sequenced and identified. Applying a well‐characterized methodological framework to the analysis of such diet samples paves the way for standardized trophic data across samples, circumventing bias introduced by visual inspection of prey remains. Moreover, molecular prey identification also decreases the per‐sample effort for dietary analysis, *i.e*., the time needed to find and identify prey remains (Oehm *et al*., 2017). Finally, the use of high‐throughput technology (*e.g*., Wallinger *et al*., 2017) for processing and analysing diet samples allows large sample numbers (*i.e*., several thousand samples, *e.g*., McInnes *et al*. 2019, Roubinet *et al*. 2018, Sint *et al*. 2019) to be examined cost‐ and time‐effectively. This is a key feature to obtain reliable and quantifiable information on trophic networks including fish both as consumers/hosts and prey as well as to examine and predict the dynamics in these food webs across space and time.

## 2 MOLECULAR ASSESSMENT OF TROPHIC INTERACTIONS: THE WORKFLOW

**FIGURE 1:**
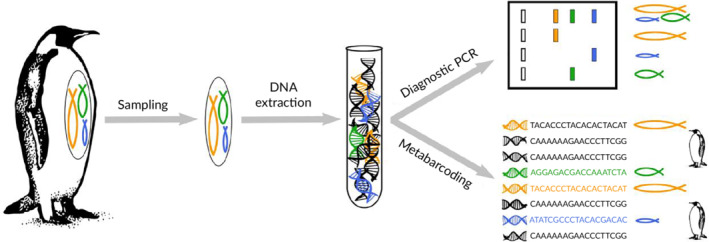
Schematic overview of the workflow when analysing dietary samples molecularly: sample collection (gut content, regurgitate, faeces), extraction of total DNA, identification of food DNA *via* diagnostic PCR and/or metabarcoding, respectively

When investigating trophic interactions molecularly (Figure 1), one of the most important things to consider is the fact that the DNA of interest, *i.e*., the DNA of the consumed food, is digested and thus will be degraded over time. Consequently, the detection of multiple copy genes, *i.e*., genes that are present in hundreds to thousands of copies per cell such as mitochondrial, chloroplast or ribosomal DNA, promotes the probability of finding semi‐digested food DNA due to the overall higher number of target molecules in the consumed tissue. Furthermore, ongoing digestion leads to fragmented DNA strands such that the detection of long DNA fragments will become increasingly difficult the longer the prey is digested (Deagle *et al*., 2006). Thus, the use of shorter DNA fragments as targets is preferred to extend the time window post consumption during which a food item can be detected (Hoogendoorn and Heimpel, 2001) and increase the overall detection probability (Keskin, 2016). It has been therefore suggested that the use of DNA fragments longer than approx. 300–400 bp should be avoided in trophic studies to maximize the detection probability (King *et al*., 2008). For the application of metabarcoding, where millions of DNA sequences are read in parallel and then matched to reference databases, this limitation to short amplicons is additionally driven by methodological constraints as only a few sequencing platforms are capable of sequencing longer amplicons. However, if specific primers are used to detect certain prey taxa, the 300–400 bp should not be seen as an absolute limit, as the overall performance of the applied assay (*i.e*., its sensitivity, which is influenced by potential mispriming, primer dimer formation, *etc*.) will also strongly influence the ability to detect food. It is thus not uncommon that a well performing assay targeting fragments of 380–480 bp can outperform a less effective one targeting 100–200 bp fragments (Waldner *et al*., 2013). If the investigated organisms are parasites or symbionts, the focal DNA is not digested and thus the length of the analysed DNA fragment is anyway of reduced importance. Nevertheless, it is also advisable to target multiple copy genes to make sure that the symbionts and parasites are detected even if only a few cells are present (Ye *et al*., 2017). If early developmental stages are to be analysed, mitochondrial genes are preferred, as eggs are especially rich in mitochondria with up to 15,000 times more organelles than can be found in somatic cells (Tourmente *et al*., 1990).

A wide range of dietary sample types can be employed in DNA‐based trophic analysis (Figure 1). For example, samples can be invasively collected by extracting the digestive part of a consumer for analysis (Braley *et al*., 2010; Jo *et al*., 2014; Marshall *et al*., 2010; Siegenthaler *et al*., 2019; Tverin *et al*., 2019) or flushing it to obtain its content, which often works well for piscivores such as penguins (Alonso *et al*., 2014). Alternatively, noninvasive sampling approaches, such as the collection of faeces and regurgitates, have successfully been applied for dietary analysis, having the advantage of minimizing the impact on the species under investigation. Noninvasive samples should be collected as fresh as possible to avoid additional breakdown of food DNA by ongoing digestion through omnipresent DNases (Palumbi, 1996), exposure to UV‐light (Deagle *et al*., 2005; Oehm *et al*., 2011) or microbial activity (Barnes *et al*., 2014). Hence faeces have been analysed to track consumption of fish (Granquist *et al*., 2018; Hong *et al*., 2019; Jarman *et al*., 2013; Sommer *et al*., 2019) and their diet (Corse *et al*., 2010; Guillerault *et al*., 2017). Additionally, pellets produced by piscivorous birds that regurgitate indigestible prey remains proofed as suitable source of trophic information (Oehm *et al*., 2017; Thalinger *et al*., 2018). Likewise, lost food items or dropped prey remains can provide valuable information on a diet. Especially at times when young are reared, prey remains are found close to nests (Bowser *et al*., 2013; Oehm *et al*., 2017).

Besides employing optimized detection assays (Figure 1), the probability to successfully identify trophic interactions *via* DNA can be enhanced by the application of high‐quality DNA isolation protocols. Such high‐end extraction protocols which are specifically tailored towards the extraction of DNA from dietary samples effectively remove potential PCR inhibitors such as phenolic compounds (Wallinger *et al*., 2017), humic acids or other complex substances known to be often present in diet samples, such as faeces (Abu Al‐Soud and Rådström, 2000; Sidstedt *et al*., 2019). Likewise, the use of high‐quality PCR chemistry and the addition of PCR enhancers for overcoming inhibition are essential to maximize food DNA detection success. Contrastingly, the often suggested dilution of DNA extracts to avoid PCR inhibition is not advised when dealing with degraded target DNA, being present only in traces (King *et al*., 2009). In such cases, a dilution of the sample could reduce the probability of detecting the food or parasite DNA.

## 3 DNA AMPLIFICATION AND CONSEQUENCES FOR FOOD DNA DETECTION AND IDENTIFICATION

Primers matching the targeted prey/parasite DNA are required for the polymerase to start the synthesis of the corresponding DNA strand, thus producing DNA molecules which can be detected and identified. While general primers match short areas of DNA which are (often not perfectly) conserved for all targeted species, specific primers are purposely designed to fit only to DNA of a certain species or taxon. There are two approaches to how the DNA of the food and/or parasites can be detected and identified: (a) diagnostic PCR and (b) diet metabarcoding (Figure 1). Cannibalistic interactions go undetected as the DNA of the consumer cannot be differentiated from that of conspecific prey with the technologies currently applied.

### 3.1 Diagnostic PCR

Assays employing specific primers are known as diagnostic PCR. They provide the possibility of discovering directly whether DNA of this taxon is present depending at the presence/absence of a specific amplicon. The merit of this type of assay is that no further analysis is necessary for answering the question of whether a specific prey has been consumed or if a specific parasite uses a fish species as a host. Apart from that, diagnostic PCR can be employed almost independently of the platform of choice (*e.g*., standard PCR, quantitative PCR, droplet digital PCR) and the potential use of fluorescently labelled probes provides the opportunity to increase assay specificity further. Multiplex PCR assays, although challenging to be developed and optimized, even allow the detection of several different food or parasite taxa simultaneously in one reaction, making diagnostic PCR a very fast and cheap way to analyse large numbers of samples (Gariepy *et al*., 2008; Harper *et al*., 2005).

Despite these advantages, so far only relatively few studies have applied diagnostic PCR to investigate fish as predator or prey (Brandl *et al*., 2015; Casper *et al*., 2007b; Keskin, 2016; Lamb *et al*., 2017). This might be due to the drawbacks of diagnostic PCR: apart from requirements regarding sufficient experience and skills for assay development, the detection is restricted to those taxa that are looked for and only a limited number of food taxa can be investigated in parallel, even when stepwise multiplex PCR approaches are applied (Thalinger *et al*., 2016).

### 3.2 Diet metabarcoding

If general food‐ or parasite‐specific barcoding primers are employed in PCR, the amplicons need to be further analysed to gain information on the consumed taxa. Usually this is done by reading the respective DNA fragment and comparing it with known DNA sequences in a reference database (DNA barcoding). Until recently, this was mainly achieved through cloning of PCR products and Sanger sequencing of individual clones (Braley *et al*., 2010; Deagle *et al*., 2007; Hardy *et al*., 2010; Leray *et al*., 2013b). Since the costs for the parallel sequencing of millions of individual DNA molecules *via* high‐throughput sequencing (HTS) approaches dropped significantly, targeted amplicon sequencing (*i.e*., diet metabarcoding) has replaced the aforementioned cloning approach. Nowadays, most trophic studies including fish as consumers or prey/host are conducted based on the metabarcoding approach (*e.g*., Bessey *et al*., 2019; McInnes *et al*., 2017b; Riccioni *et al*., 2018; Schwarz *et al*., 2018; Waraniak *et al*., 2019). Even the combination of several general barcoding primer pairs is possible, to cover a very wide food spectrum of generalist consumers (Deagle *et al*., 2009). The application of general barcoding primers has the advantage that no or very little *a priori* knowledge on potential food is needed to elucidate the diet of a species and also unexpected food sources might be detected (Leray *et al*. 2013). However, this comes at the cost that the primers often also amplify the consumer DNA. If consumer DNA is overrepresented compared to prey DNA in a sample, this can lead to a dramatic drop in the number of informative diet DNA sequences obtained by metabarcoding (Krehenwinkel *et al*., 2017). Hence, the application of blocking primers, which can prevent or reduce the amplification of a certain taxon (*e.g*., the consumer), is particularly useful for dietary studies (Su *et al*., 2018; Vestheim and Jarman, 2008). A metabarcoding study on the gut content of stickleback (Jakubaviciute *et al*., 2017; Leray *et al*., 2013a) demonstrated that blocking primers should be preferentially used over restriction digestion for predator DNA removal as they recover greater prey diversity. However, potential co‐blocking of some food taxa or shifts in read numbers across taxa can be a problem (Piñol *et al*., 2015). If consumer and prey are closely related, as it is the case in piscivorous fish, their DNA sequences might be similar in the target region, increasing the risk for unwanted effects.

### 3.3 Comparison of diagnostic PCR and metabarcoding

There are advantage and drawbacks in both the two basic principles for molecular diet analysis and parasite detection, and the decision of which method is better suited strongly depends on the research question asked (Table 1). Provided the specific primers employed in diagnostic PCR are well designed, the results of these assays are usually very robust and reproducible (Rennstam Rubbmark *et al*. 2019). This is because the ability to detect a certain prey/parasite taxon is barely influenced by the presence or absence of other DNA types such as the consumer, other prey or microbes (Sint *et al*., 2012). Only close to the detection limit, which can be below 20 molecules per PCR (Thalinger *et al*., 2016), can variability in prey/parasite DNA detection success be observed (Sint *et al*., 2012). This effect depends mainly on whether the primers managed to attach to the few template molecules during the first few PCR cycles (Ruano *et al*., 1991).

**TABLE 1:** Comparison of diagnostic PCR and metabarcoding for assessing trophic interactions *via* the detection and identification of food DNA

	Diagnostic PCR	Metabarcoding
*A priori* knowledge needed	Yes	Little
Primer type	Taxon‐specific	General or group‐specific
Investigated prey spectrum	One or few taxa	Potentially whole diet
Research questions	Functionally important prey taxa	General prey spectrum
Assay development	Can be challenging (multiplex PCR)	Many published assays
Interference with consumer DNA	Low	Can be high
Time requirement for analysis	Low	High
Suitability for high sample numbers	High	Medium
Bioinformatics	No	Yes

General barcoding primers, such as those used for metabarcoding, however, will likely have an imperfect match to some of the taxa targeted. While still being able to bind to and amplify these DNA strands, the efficiency can be strongly reduced, dependent on the number and location of the mismatches at the priming sites, leading to biased amplification of the different species being present in a biological sample (Piñol *et al*., 2015). Even if a perfect match is realized for all potential targets, the base composition or number of template molecules of an individual target can influence the amplification simply by stochastic mechanisms. This means that abundant types of DNA are more likely to be amplified than rare ones, or certain DNA sequence variants are preferentially amplified and thus sequenced over others (Kelly *et al*., 2019; Ruano *et al*., 1991). This stochasticity plays an important role in the limited repeatability of metabarcoding results (Rennstam Rubbmark *et al*., 2019) and shows that metabarcoding approaches, while highly suited to describe the general prey spectrum, are often not the best choice to detect rare targets. Moreover, closely related species often differ only by a few bases in the target genome region, which can make it challenging to distinguish the actual presences of these two species from a PCR or sequencing error. Furthermore, the ability to identify the diet sequences derived by metabarcoding depends on the sequence reference databases used. Depending on the organisms of interest and the target genes, several public databases [*e.g*., GenBank (www.ncbi.nlm.nih.gov/genbank/), BOLD (www.boldsystems.org), SILVA (www.arb‐silva.de) or MitoFish (http://mitofish.aori.u‐tokyo.ac.jp)] can be used to identify the obtained sequences. However, taxonomic coverage in such databases and consequently the resolution of dietary composition can vary greatly across genes, taxa and the geographic region of interest (Weigand *et al*., 2019). Additionally, databases potentially contain misidentified DNA sequences, or sequences identified *via* “reversed taxonomy” (*i.e*., *via* DNA barcoding and not morphology). Still, the majority of taxonomic identifications seem to be correct (Leray *et al*., 2019), such that adverse effects for dietary studies may be unlikely. This calls for a thorough preassessment to evaluate which taxonomic resolution can be obtained by using public reference databases. Therefore, it is not surprising that many publications rely on custom curated databases containing only verified sequences of plausible species in the investigated geographic range to improve the fit and taxonomic accuracy of the results (*e.g*., Hanfling *et al*., 2016). There are many different tools and strategies for processing raw metabarcoding reads until they are matched against a reference database. Some applications are specific to certain processing steps (*e.g*., “Cutadapt”) and many researchers develop their own pipelines using various tools. Other applications, such as “obitools”, “QIIME2”, “SLIM” and “JAMP”, provide a framework for all these individual steps to facilitate sequence processing for users. Addressing the many aspects necessary for successful diet metabarcoding and subsequent sequence analysis comprehensively goes beyond the scope of this article, but has been dealt with elsewhere (*e.g*., Alberdi *et al*., 2018; Deagle *et al*., 2019; Pompanon *et al*., 2012).

### 3.4 The importance of primers

**FIGURE 2:**
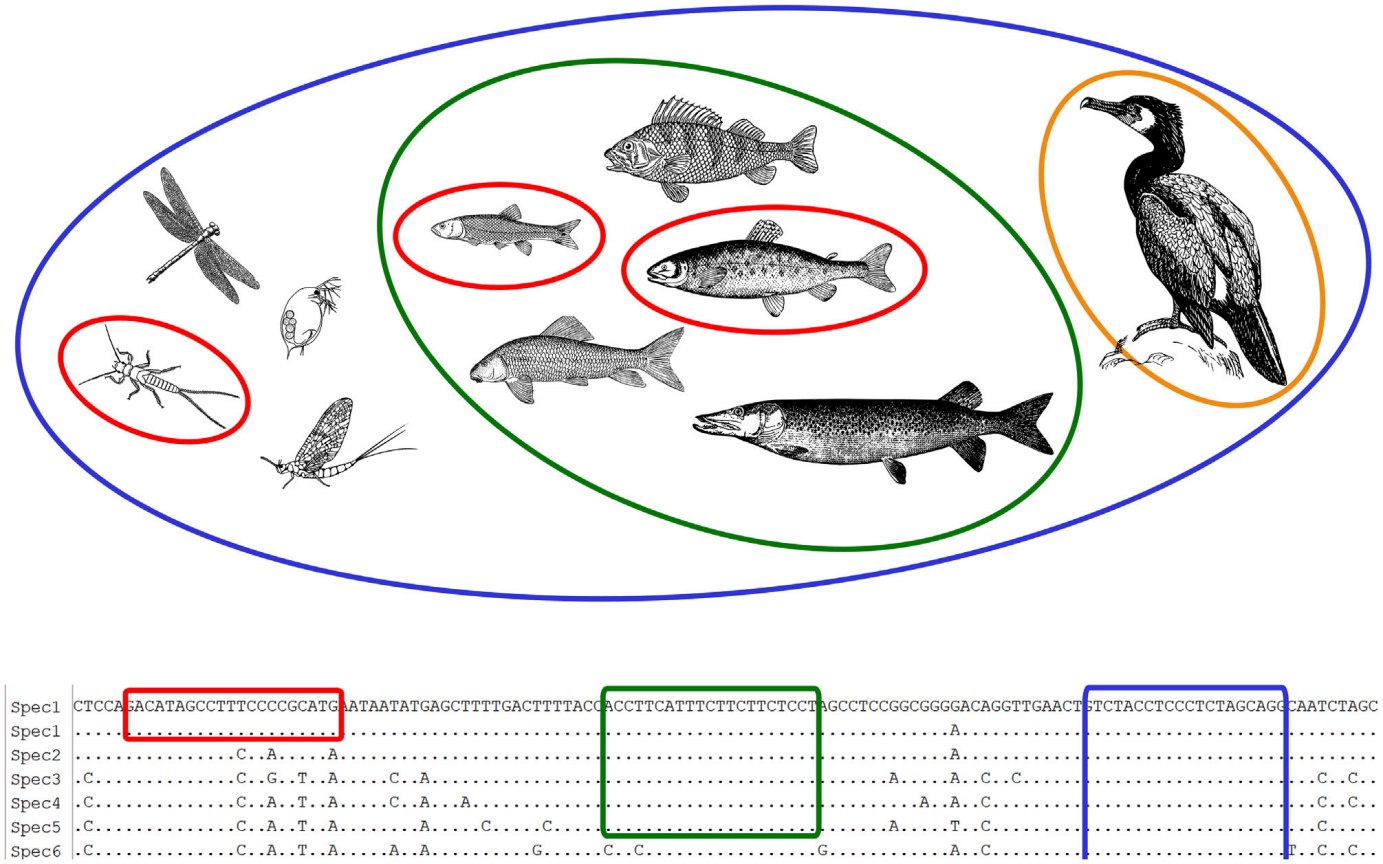
Schematic overview of different types of primers: species‐specific (red), group‐specific (green), general (blue) and blocking (yellow) primers in a hypothetical food chain and corresponding DNA sequences of involved taxa. Dots in the sequence alignment denote identical bases as in the topmost sequence

The design and selection of PCR primers needs considerable care and the requirements for such primers differ between the two basic approaches of molecular diet analysis (Figure 2). For diagnostic PCR, a species or taxon/group specific primer pair is required, meaning that the binding sites for the primers need to be conserved within the targeted taxon. At the same time, it must be different to all nontarget taxa to prevent the amplification of their DNA, which would otherwise result in false‐positive detections. For metabarcoding approaches on the other hand, not only the primer binding sites need to be considered, but also the resulting amplicon length needs to be suitable for metabarcoding. Depending on the HTS platform used, this can vary between less than 100 and several hundred base pairs (Ergüner *et al*., 2015). Finally, the selected DNA region needs to provide stretches that are conserved among taxa (to place the primers a suitable distance apart), but the sequence information between the primer binding sites needs to be variable enough to allow taxon identification based on these sequences (Clarke *et al*., 2014).

## 4 QUANTIFICATION OF FOOD DNA

Due to the co‐occurrence of consumer DNA, measuring total DNA concentration in a dietary sample is not informative regarding the amount of food or parasite DNA. Even so, several methods exist to quantify this specific target DNA (Table 2). As the concentration of food DNA is typically not high in dietary samples, standard PCRs are usually terminated before reaching product saturation (Ruano *et al*., 1991). Thus, the amount of amplicon being generated correlates well with the amount of template DNA (Thalinger *et al*., 2019). The application of prey‐specific diagnostic CE‐PCR (*i.e*., capillary electrophoresis PCR, which stands for “diagnostic PCR” combined with fragment analysis based on capillary electrophoresis) provides an easy way for relative comparisons of prey/parasite DNA concentration in different samples based on the signal strength obtained. If several assays are balanced in their amplification efficiency (Sint *et al*., 2012), even comparisons across distinct prey types are possible. Similar information can be gained *via* qPCR and standard curves to relate the signal back to the starting concentration of template DNA (Bowles *et al*., 2011). When digital PCR is applied, the reaction is split into thousands of individual subreactions and the outcome of each of those is evaluated separately. Digital PCR enables absolute quantification of the starting DNA concentration based on statistical approximations. Whether (relative) abundances of read numbers from metabarcoding can be used as a measure for DNA concentrations is still strongly debated and cannot, due to the numerous factors influencing PCRs, sequencing and bioinformatic analysis, be generalized (Alberdi *et al*., 2018; Kelly *et al*., 2019; McLaren *et al*., 2019; Piñol *et al*., 2015). Feeding experiments showed, for example, that relative proportion of read numbers can strongly deviate from the known amount of consumed fish (Deagle *et al*., 2010, 2013). As the reads for certain species were constantly over‐ or underestimated, attempts were made to correct for this systematic error *via* the application of correction factors, for example based on the lipid content of the fish (Thomas *et al*., 2014). The question of how the amount of food DNA relates to the mass and/or number of diet items consumed and how this information can be transformed into dietary data is still under debate (see Deagle *et al*. (2019) for a review on the metabarcoding side of this question).

**TABLE 2:** Overview of DNA quantification methods and their potential applications

	Total DNA within extract^a^	DE‐PCR	CE‐PCR	qPCR	dPCR	HTS
Co‐measurement of consumer DNA	Yes (but no separation from other DNA)	No	No	No	No	Yes (separation)/no^b^
Quantification of PCR amplicon	No	Yes (rough estimate)	Yes	Yes	No	Partly^c^
Quantification of starting template	No	No	Yes, estimation at low concentrations *via* standard curve	Yes, estimation *via* standard curve	Yes, absolute quantification	No
Definition of detection threshold	No	No	Yes	Yes	Yes	Yes
Comparisons across consumers	No	No	Yes	Yes	Yes	Correction factors needed
Comparison across prey taxa	No	No	Yes, if balanced	Yes, if balanced	Yes, if balanced	Correction factors needed

^a^ Measured using a spectrophotometer or a fluorescent dye.

^b^ Dependent on consumer DNA amplification of primers.

^c^ Often more than one PCR conducted, additional clean‐up steps and sequencing can affect results.

*Note*. DE‐PCR, diagnostic endpoint PCR with gel electrophoresis; CE‐PCR, diagnostic endpoint PCR with capillary electrophoresis; qPCR, quantitative real‐time PCR; dPCR, digital PCR; HTS, high throughput sequencing (= metabarcoding). The methods cannot be compared among each other easily regarding the cost and time needed to process a specific number of samples as both depend on the type of reagents used, personnel costs and the laboratory equipment available (especially high‐throughput formats), all of which can vary significantly among regions, suppliers and for specific products. Generally, costs for consumables per sample can be expected to be lowest for the measurement of the total DNA concentration and increase gradually from DE‐PCR, CE‐PCR, qPCR and dPCR towards HTS. In terms of handling time per sample, CE‐PCR represents the most effective method among the PCR approaches as it can be multiplexed and the electrophoresis automated.

## 5 INTERPRETATION OF MOLECULAR TROPHIC DATA

### 5.1 Conversion of detected diet DNA to trophic data

**FIGURE 3:**
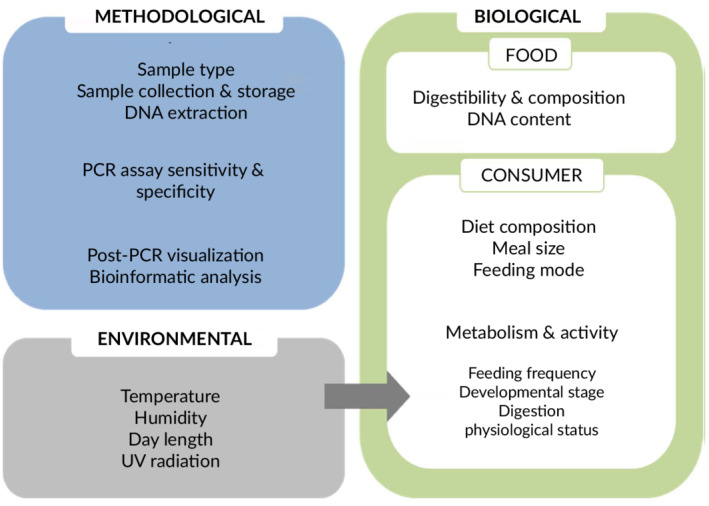
A range of factors affect the fate of food DNA in dietary samples and influence the interpretation of molecularly derived trophic data. These factors can be grouped into methodological, biological and environmental aspects

When interpreting molecularly derived trophic data various factors need to be considered comprising environmental, biological and methodological aspects (Figure 3). There are two types of molecular prey detection data: (a) presence/absence data and (b) quantitative data. Independent of the way the data were obtained (*via* diagnostic PCR, real‐time qPCR or metabarcoding), presence/absence data always require a detection threshold to be set, *i.e*., a measurable parameter such as a fluorescence threshold (Jensen *et al*., 2018; Thalinger *et al*., 2016), or an absolute or relative number of target reads (Deagle *et al*., 2019; Thomas *et al*., 2017). Presence/absence data are generally used to calculate frequency of occurrence (*i.e*., the number of samples containing a specific prey taxon; FOO), which is often displayed in percent (F% or %FOO) or rescaled to per cent of occurrence (POO), providing a summed up occurrence rate of 100% across all prey taxa (Baker *et al*., 2014; Deagle *et al*., 2019). Such semiquantitative estimates give a good proxy for the strength of specific trophic interactions. These metrics can be calculated from data obtained by diagnostic PCR, metabarcoding and morphological analyses alike, providing good comparability between studies. Quantitative or semiquantitative data provide absolute or relative measures of target DNA. They can be received from both quantitative types of diagnostic PCR (Table 2) in the form of (absolute) target DNA copies per microlitre (Bowles *et al*., 2011; Deagle and Tollit, 2007; Jensen *et al*., 2018) or (relative) quantification of CE‐PCR signal strength between taxa (Thalinger *et al*. 2016), and metabarcoding in the form of relative read abundance (RRA) accounting for varying sequencing depths between samples (Ford *et al*., 2016; McInnes *et al*., 2017b; Thomas *et al*., 2017). Generally, taxon‐specific diagnostic PCR results are more straightforward to interpret as samples undergo only a single amplification and have been shown to provide quite good estimates of fish consumption by piscivores (Bowles *et al*., 2011; Deagle and Tollit, 2007). Contrastingly, most of the recent fish dietary studies rely on metabarcoding approaches and RRA. Considerable effort has been put into identifying and accounting for amplification, sequencing, and bioinformatic biases which metabarcoding samples are exposed to (Lamb *et al*., 2017; Zinger *et al*., 2019).

Although there exist several options for quantifying food DNA, translating the amount of diet/parasite DNA to the amount of prey consumed is not trivial and has to be evaluated separately. In this context, it has to be taken into account that apart from dependencies on the targeted genes (single *vs*. multiple copy and mitochondrial/chloroplast *vs*. nuclear) or varying copy numbers of DNA in different tissues of an organism (Herbers *et al*., 2019), numerous biological and environmental factors influence the final number of food DNA molecules present in a dietary sample (Figure 3). Therefore, the interpretation of the different qualitative and quantitative measures and their usefulness for assessing trophic relations between species has been controversially discussed. On the one hand, Deagle *et al*. (2019) show that %FOO overestimates the importance of rare prey items in metabarcoding studies, leading to potential pitfalls for analyses on bioenergetics and niche partitioning. Therefore, research addressing the foraging ecology of fish and piscivores beyond prey diversity tends to employ a mixture of molecular and morphological methods to obtain a holistic picture of feeding ecology (Alonso *et al*., 2014; Thalinger *et al*., 2018; Thomas *et al*., 2017). On the other hand, Baker *et al*. (2014) argue in favour of %FOO since it makes comparisons between studies easy and because other measures applied in studies involving morphological fish prey identification are more prone to introduce other biases. Which type of quantitative measure is best to be used will depend on the predator–prey system studied and the characteristics of the molecular methodology employed.

### 5.2 Consumer and prey‐specific post‐feeding detection intervals and gut transition times

For the interpretation of molecularly detected prey within field‐derived diet samples and for the design of efficient field sampling campaigns, it is crucial to have a basic understanding of digestive processes and gut transition times of the targeted consumer to estimate the timeframe of consumption and original meal size (Moran *et al*., 2016; Thalinger *et al*., 2017). Depending on the nature of the dietary sample, three different timeframes need to be considered. First, for stomach samples obtained *via* dissection (*e.g*., Hunter *et al*. 2012) or flushing (Alonso *et al*., 2014) the time a prey item remains in the digestive tract is of interest. Second, for faecal samples, gut transition time and the number or fraction of individual meals contained in one defecation should be known (Bowles *et al*., 2011; Casper *et al*., 2007b; Deagle *et al*., 2005; Thalinger *et al*., 2017). Finally, in studies relying on regurgitates such as pellets, the frequency of pellet production is important and the likelihood of detecting a prey item consumed within this interval needs to be determined (Oehm *et al*., 2017; Thalinger *et al*., 2017). Independent of the sample type and study system, calibratory feeding experiments associated with trophic field studies provide important information on food DNA detection for specific consumers and prey (King *et al*., 2008). Parameters commonly reported in feeding trials are minimum time passed until the first detection of a prey item, the maximum detection time (Casper *et al*., 2007b; Thalinger *et al*., 2017) and the timepoint post consumption at which a prey item has a 50% detection probability (often also referred to as the detectability half‐life, see Greenstone *et al*. (2014) for a summary of the nomenclature used to describe this point). Moreover, such feeding experiments allow the impact of various factors to be investigated separately, as, for example, consumer and prey identity, meal size, diet composition or ambient temperature (Albaina *et al*., 2010; Thalinger *et al*., 2018; Thomas *et al*., 2014) (see also Figure 3). First, consumer‐related variables such as species identity and life stage can play an important role (Greenstone *et al*., 2010; Sint *et al*., 2018). Second, prey‐related factors leading to differential digestion (*i.e*., different digestability) and amplification biases in molecular analyses (Thomas *et al*., 2014) or meal size can influence prey detection. For example, a small amount of target DNA in a gut content sample can either result from a small meal that has been digested for a short period, or from a large meal being digested over an extended time span (Thalinger *et al*., 2017). Third, environmental variables such as ambient temperature are known to influence digestion rates (Carreon‐Martinez and Heath, 2010; Hunter *et al*., 2012). Fourth, the level of standardizing meal size varies between consumer groups: whilst it is possible to force‐feed fish with standardized meals (Hunter *et al*., 2012), this is generally not possible and/or permitted for captive piscivorous birds and mammals for which standardized prey uptake is possible but not guaranteed (Bowles *et al*., 2011; Thalinger *et al*., 2017; Thomas *et al*., 2014). Additionally, starvation of consumers during feeding trials is usually prohibited, making it impossible to determine the effect of an individual meal independent of latter food uptake (*i.e*., chaser prey) (Greenstone *et al*., 2014; Thalinger *et al*., 2017). Finally, the power of such feeding trials is often limited by the small number of consumers available (*e.g*., avian and mammalian piscivores) and they are only valid if gut transition time and prey consumption do not differ significantly between captive and wildlife individuals (Bowles *et al*., 2011; Casper *et al*., 2007b; Deagle *et al*., 2005; Thalinger *et al*., 2016, 2017; Thomas *et al*., 2014).

Experiments regarding fish as consumers have so far primarily focused on digestion in their guts influencing the detectability of small prey items such as fish eggs or larvae from stomach samples; in such feeding trials it was found that prey detection success starts decreasing already more than 2 h post feeding and maximum detection times range between 24 and 48 h (Carreon‐Martinez and Heath, 2010; Hunter *et al*., 2012; Waraniak *et al*., 2019).

Far more feeding trials focus on fish as prey. Overall, they reveal a broad variety in the time frame of prey detection post feeding between different piscivores: in faeces of the Eurasian otter, fish prey was only detected in samples collected 24 h post consumption (Thalinger *et al*., 2016), for seals and sea lions (Casper *et al*., 2007a; Deagle *et al*., 2007) initial detections were present at about 7–8 h post consumption and final detections at about 32–48 h, although it needs to be noted that the estimation of the defecation time was challenging (sampling impossible during the night). Two studies (Bowles *et al*., 2011; Deagle *et al*., 2005) found a good correlation between the amount of fish consumed and the number of prey DNA molecules amplified, with small meals being reliably detected. In a study on cormorants, earliest prey detection in faeces was reported 1.5–2.5 h post feeding and the maximum prey detection time in faeces was 75 h post feeding (Thalinger *et al*. 2017). In this case, however, small meals were not reliably detected and showed significantly lower detection probabilities across time than large meals.

### 5.3 Food chain errors due to secondary predation

The food DNA detected in a dietary sample can either come directly from the food eaten by the consumer, the so‐called primary prey, or stem from secondary prey, *i.e*., the food DNA which is contained in the digestive system of the primary prey (Sheppard *et al*., 2005). Secondary predation (also called hyperpredation) has received far too little attention in dietary studies of fish, albeit the topic is highly relevant and often addressed in review articles assessing the potential of (molecular) methods to study predator–prey relations and associated pitfalls (Deagle *et al*., 2019; Sousa *et al*., 2016). In practice, there are different ways of accounting for secondary predation during the interpretation of dietary field data. Often, taxa which are known to not be preyed upon are removed from the dataset prior to analysis (*e.g*., fungi, macroalgae and Chromista are not fed upon by sticklebacks) and taxa which are suspected to be derived from secondary predation (*e.g*., copepods in the diet of Adélie penguins) are addressed in the discussion (Jakubaviciute *et al*., 2017; Jarman *et al*., 2013). Additionally, the detection of diet taxa suspected to be secondarily predated is checked for consistent co‐occurrence with suitable meso‐predators in the dataset, indicating the potential of secondary predation, and in case of unique detections, suggesting direct ingestion (Hardy *et al*., 2017; McInnes *et al*., 2017b; Merten *et al*., 2017; Raso *et al*., 2014). Another opportunity is the combination of molecular and morphological approaches, providing the possibility of ruling out secondary predation by comparing hard‐part‐based size estimations between the potential primary and secondary prey items. Accordingly, Oehm *et al*. (2016) were able to reduce the potential for food chain errors by 80% when analysing prey remains in the digestive tracts of cormorants. If the study design is flexible enough, dietary samples of both the top predator and the primary prey species can be analysed with similar molecular methods, as done by Bowser *et al*. (2013) for Atlantic puffins and Atlantic herring. This led to the detection of plankton prey in both herrings (primary consumption) and puffins (secondary consumption). These results highlight the need to carefully consider secondary predation. Future studies would benefit from a consistent way of addressing this issue. If there is enough information on predators and their prey species within the examined food web, compiling the results twice, once excluding all potential secondary prey items and once including all prey detections, could be a viable solution.

### 5.4 Detection of nonfood DNA

The application of metabarcoding in dietary studies of generalist consumers enables the detection of high levels of biodiversity from dietary samples (*e.g*., Bessey *et al*. 2019). For example, more than 1000 operational taxonomic units were detected in brown shrimp stomach contents by Siegenthaler *et al*. (2019) and compared to the biodiversity detected in sediment and water samples from the same environment. When generalist consumers are investigated using general primers, certain precautions against contamination should be taken during sampling to avoid detection and misinterpretation of environmental DNA as food DNA. When faeces or regurgitates are used, surface contamination stemming from the location at which the dietary sample was deposited should be considered, for instance by providing artificial surfaces and exchanging them between sample collections (Oehm *et al*., 2017), or by only collecting samples from decidedly “clean” surfaces (McInnes *et al*., 2017a). In case consumers are handled to obtain stomach samples (Bessey *et al*. 2019, Siegenthaler *et al*. 2019), it is advisable to consider body‐surface contamination as well. In studies conducting dietary analyses of terrestrial arthropods, body‐surface contamination has been repeatedly addressed since 2010, for example Remén *et al*. (2010) found washing and dissection of oribatid mites to be necessary and Greenstone *et al*. (2012) recommended commercial bleach (sodium hypochlorite) for removing surface contamination without affecting prey DNA detection success. Staudacher *et al*. (2013) and Briem *et al*. (2018) furthermore applied and refined surface bleaching procedures for dietary studies of click beetle larvae and spotted wing drosophila, respectively. For dietary studies based on stomach samples of aquatic species, environmental contaminations are rarely addressed despite a general emphasis on “good laboratory practice” (King *et al*., 2008). The processing of lobster larvae described by O'Rorke *et al*. (2012), who promote the use of a surface swab as control and the use of disposable materials for gut content sampling, is one of the few counter‐examples. There is also a detailed discussion on the influence of surface contamination during beachside sampling of Mobula ray stomachs by Bessey *et al*. (2019).

Metabarcoding of putative dietary DNA is especially prone to pick up such environmental DNA (eDNA; Thomsen and Willerslev, 2015) and several studies from fish‐based food webs demonstrate this issue: in a metabarcoding dietary analysis of coral‐dwelling predatory fish Leray *et al*. (2015) identified 292 OTUs (51% of these with species‐level matches) in the gut contents of the arc‐eye hawkfish (*Paracirrhites arcatus*), the flame hawkfish (*Neocirrhites armatus*) and the coral croucher (*Caracanthus maculatus*), collected at the Moorea atoll (French Polynesia). The authors concluded that the three species show a highly partitioned, generalist diet with nearly 80% of prey items consumed by only one fish species. However, most of the taxa consumed by a single species occurred only at low read numbers and the question arises of how strongly the putative diet separation is biased by eDNA amplified from the stomach samples rather than DNA stemming from focused dietary interactions. Similarly, the exceptionally broad diets of fish species compared to previous knowledge based on morphological prey remains reported in other studies (Jakubaviciute *et al*., 2017; Riccioni *et al*., 2018; Sousa *et al*., 2016) might be fraught with the same problem of mixing eDNA and food DNA, providing methodologically inflated food spectra.

### 5.5 Scavenging of food

Another aspect involving the possibility for confusion in both molecular and nonmolecular diet analysis is scavenging. Per definition, scavenging is the consumption of already dead food, contrary to predation, which includes its killing. However, the DNA analysis of food present in the gut of the consumer does not allow differentiation between active predation and scavenging (Foltan *et al*. 2005). Similar to live food, the DNA of both carrion and decayed plant food can be detected at extended times post feeding (Juen and Traugott, 2005; Wallinger *et al*., 2013). As a result, molecular diet analyses may lead to an overestimation of the impact of predators on prey populations if primarily carrion is scavenged. Whether the differentiation of these two feeding strategies is important for a study depends on the questions asked. For the energy flow in a food web and the supplement of consumers it may be a minor factor whether the consumed prey had been dead or not. Wilson & Wolkovich (2011) recently highlighted that scavenging links have been underestimated in food webs ranging from marine to terrestrial ecosystems, and that substantially more energy is transferred per scavenging link than per predation link. When it comes to the investigation of regulatory management, it is highly relevant whether a predator was actively hunting and killing its prey or taking advantage of an already dead animal. In terrestrial ecosystems, especially when dealing with agricultural systems, pest control and thus the direction of regulation pathways (bottom up *vs*. top down) are important to know. Here, the differentiation of predation and scavenging can be essential (von Berg *et al*., 2012). The situation might be very similar when it comes to the assessment of the loss of fish *via* piscivores and their potential as food competitors for humans. However, in contrast to terrestrial ecosystems, only a few comparable studies exist in aquatic systems. McInnes *et al*. (2017b) considered scavenging of albatrosses close to commercial fishing vessels, while (Leopold *et al*., 2015) defined parameters to differentiate wounds on stranded harbour porpoises stemming from grey seal attacks on living or already dead porpoises. Knowing the feeding behaviour of the animals studied is of great importance when interpreting dietary data, for example van der Reis *et al*. (2018) analysed the diet of the New Zealand scampi (*Metanephrops challengeri*) *via* metabarcoding, suggesting that this deep sea lobster has a broad diet spectrum, with a high reliance on scavenging a diverse range of pelagic and benthic species from the seafloor. Although scavenging plays a similar functional role in terrestrial and aquatic food webs, Beasley *et al*. (2012) suggested that several fundamental differences do exist. In particular, the movement of carcasses in marine ecosystems (*e.g*., wave action, upwelling and sinking) diffuses biological activity associated with scavenging and decomposition across large, three‐dimensional spatial scales, creating a unique spatial disconnect between the processes of production, scavenging and decomposition, which in contrast are tightly linked in terrestrial ecosystems.

## 6 APPLICATION OF DNA‐BASED DIET ANALYSIS TO DETERMINE FISH AS PREY AND THE PREY OF FISH

Molecular techniques have been applied in different ways to examine the diet of fish and to determine which fish species are consumed by piscivores. The simplest but also most straightforward applications in this context are diagnostic PCR assays, which allow the detection of particular prey taxa (single species or larger taxonomic groups of organisms) within diet samples. For example, Jensen *et al*. (2018) employed a species‐specific qPCR system to examine the gut content of 17 mesopelagic fish taxa collected in the Sargasso Sea for DNA of larvae of the critically endangered European eel, *Anguilla anguilla*. Overall, 10% of the investigated specimens contained DNA of *A. anguilla*, motivating further studies on how the European eel is utilized as prey in this oceanic ecosystem and what this implies for the future of this fish species. In contrast, Lamb *et al*. (2017) employed a set of group‐specific primers which amplify the mitochondrial DNA of cnidarian jellyfish species occurring in the Irish Sea within a diagnostic PCR assay to assess the role of jellyfish as prey in marine food webs. The authors show that jellyfish predation may be more common than previously acknowledged, with jellyfish DNA detected in 27.6% and 11.6% of herring and whiting stomachs, respectively, representing the two most abundant fish species with the highest detection frequencies of jellyfish prey in this study. This work refutes the notion that jellyfish predation is rare and demonstrates the value of such comparably simple molecular detection systems to improve the understanding the trophic role jellyfish play in ecosystems and in predicting jellyfish blooms.

Diagnostic PCR assays can also be used to assess the consumption of multiple food types within one assay when primers for multiple prey targets are multiplexed (Harper *et al*., 2005; Sint *et al*., 2012). Employing seven species‐specific primer pairs in three multiplex PCR assays, Bade *et al*. (2014) tested the digestive tract samples of the Cownose ray (*Rhinoptera bonasu*) for DNA of commercially important bivalve species. The authors found that the rays ate stout tagelus and soft‐shell clams but there was no evidence of consumption of commercially important oysters, hard clams and bay scallops. Another example is the extensive multiplex PCR system, allowing for the stepwise testing of diet samples for DNA of 31 Central European freshwater fish species, which was presented by Thalinger *et al*. (2016). This system comprises six PCR assays that were used to detect preyed fish in European otter spraints and kingfisher droppings as well as faeces and pellets of cormorants (Oehm *et al*., 2017; Thalinger *et al*., 2018).

Classical DNA barcoding, *i.e*., the amplification and sequencing of DNA fragments to identify species (Hebert *et al*., 2003), can also be used to identify prey remains which are retrieved from diet samples. This is extremely helpful for those parts which cannot be identified by visual means as they lack diagnostic morphological features. Such an approach was employed by Aguilar *et al*. (2017), who identified semidigested piscine prey remains in the gut content of the invasive blue catfish, *Ictalurus furcatus*, the non‐native, but established Channel catfish, *Ictalurus punctatus*, and the native white catfish, *Ameiurus catus*, in Chesapeake Bay, USA. Employing morphological identification, only 9.4% and 12.1% of the prey remains could be identified to species and family level, respectively. In contrast, the addition of DNA barcoding allowed for 91.6% species identification of the piscine prey remains, representing 23 species and including several endangered ones for which restoration efforts are ongoing. The authors concluded that DNA barcoding of degraded prey items was found to be an effective approach for strengthening the resolution of trophic analyses, including diet comparisons among sympatric native and non‐native predators. Likewise, a DNA barcoding approach of prey remains was employed in a study on parasite catfishes (Trichomycteridae), which show a mucophagic, lepidophagic or hematophagic feeding mode, making the host identification *via* conventional diet analysis practically impossible (Bonato *et al*., 2018). DNA barcodes generated from mucus and scale samples dissected from the digestive tract of the catfish revealed that Characiformes are their preferred hosts, revealing, for the first time, the host species of parasitic fish bearing mucophagous habits under natural conditions.

The application of metabarcoding in diet analysis has opened exciting new possibilities to assess the diet of animals in very broad taxonomic scales (Pompanon *et al*., 2012). In fact, the first study employing this powerful approach was conducted in a marine system where the colony‐wide diet of Australian fur seals (*Arctocephalus pusillus doriferus*) was examined, identifying 54 bony and four cartilaginous fish as well as four cephalopod species as their prey (Deagle *et al*., 2009). Metabarcoding has also been employed to assess the diet of different fish species, for example Jakubaviciute *et al*. (2017) studied the diet of stickleback in the western Baltic Sea coast, using both DNA metabarcoding and visual analysis of stomach contents. Overall, 192 stickleback individuals were examined, revealing 120 prey taxa belonging to 15 phyla, 83 genera and 84 species, an unusually high prey diversity compared to the outcomes of previous studies. DNA metabarcoding has also been used to examine the diet of invasive fish: within the stomach contents of 63 lionfish (*Pterois volitans*) collected inshore and offshore reefs of La Parguera, Puerto Rico, 39 fish species were found to be consumed (Harms‐Tuohy *et al*., 2016). Metabarcoding of faecal samples collected from European catfish (*Silurus glanis*) identified a broader diet than conventional stomach content analysis (Guillerault *et al*., 2017), with the added advantage that this examination was noninvasive.

Apart from the identification of prey in fish, diet metabarcoding has also been widely used to examine the diet of piscivorous birds (*e.g*., Horswill *et al*., 2018; Kleinschmidt *et al*., 2019; Komura *et al*., 2018; McInnes *et al*., 2017b), pinnipedes (*e.g*., Berry *et al*., 2017; Hardy *et al*., 2017; Peters *et al*., 2015; Thomas *et al*., 2017) and nonvertebrate fish consumers (*e.g*., O'Rorke *et al*., 2012). Despite the continued enthusiasm for the manifold possible applications of this powerful approach, care needs to be taken as how the compiled data is to be bioinformatically analysed and interpreted to obtain meaningful trophic data sets (see below).

The combination of the information on both prey and consumer DNA has been utilized to assess sex‐specific dietary choices. Schwarz *et al*. (2018) examined faeces of male and female harbour seals (*Phoca vitulina*) in Canada, identifying prey DNA *via* metabarcoding and using qPCR to determine the sex of the seals. The novel combination of molecular techniques demonstrated that diet differences between males and females were consistent across sites and years. This suggests that seals differ in their foraging strategies depending on their sex, indicating larger impacts on salmonids by males than females. Molecularly sexed regurgitated cormorant pellets in the German Alpine foreland indicated the same trend (Thalinger *et al*., 2018). The dietary information was retrieved by a combination of prey hard part analysis (allowing counting and estimating of the size of the prey fish) and DNA‐based prey identification. In this case, male diet was characterized by higher prey diversity and the consumption of larger fish, albeit prey composition did not indicate the use of different water bodies by the sexes.

## 7 CHALLENGES AND OUTLOOK

DNA‐based approaches offer substantial advantages compared to nonmolecular techniques for unravelling trophic links between fish, their prey and their predators. Nevertheless, there are also challenges and limitations which need to be considered when implementing DNA‐based food web studies.

The first challenge is related to the technological setup. Although facilities for conducting DNA analyses in general are widely available, these laboratories often do not meet the specific demands for molecular diagnostics at the levels necessary for diet analysis. Ideally, an isolated clean room laboratory is available for DNA extraction and assay preparation (*i.e*., all pre‐PCR work), whilst all post‐PCR work is conducted in a spatially separated laboratory to avoid introducing contamination of the samples and assays *via* DNA carryover, thereby generating false‐positive detections. In fact, molecular trophic diagnostics need to be executed with specific standards and work flows similar to work with other environmental DNA (eDNA), ancient DNA (aDNA) or forensic DNA (fDNA) (Alaeddini *et al*., 2010; Deiner *et al*., 2017; Thomsen and Willerslev, 2015). In comparison to eDNA, aDNA and fDNA, where usually only few samples are analysed, trophic studies often entail the assessment of much larger sample numbers ideally ranging between hundreds and thousands of samples. This means that besides the provision of a high‐quality diagnostic laboratory, high‐throughput technology and laboratory routines need to be implemented for processing large sample numbers in a cost‐ and time‐effective manner. Molecular diagnostic work also demands standardized sample processing to avoid introducing variability due to differently treated samples, for example when generating DNA extracts or conducting PCRs. Indeed, these are two of the major structural hurdles the field of DNA‐based analysis of trophic interactions has to cope with, a topic rarely acknowledged in the literature at all (but see Kitson *et al*. 2019, Wallinger *et al*. 2017). In fact, the power of trophic data which is based on the assessment of dietary samples, using both molecular and nonmolecular approaches, is strongly correlated to the number of samples analysed, *i.e*., sufficiently large sample numbers are needed to statistically assess the effect of environmental changes on trophic links and/or to compare consumers with regard to their trophic ecology. Regarding fish as predator and prey, so far only a small number of DNA‐based studies have built their analyses on sufficiently large data sets, which hampers their actual impact for obtaining a comprehensive understanding of fish‐based food webs.

The rapidly evolving field of molecular trophic analysis has opened up a wide field of varieties but also led to increasing methodological complexity (*e.g*., Alberdi *et al*., 2018). For example, the establishment of new diagnostic PCR assays from scratch entails the often cumbersome development of appropriate primers meeting all requirements regarding specificity and sensitivity. Diet‐related metabarcoding work, however, needs to deal with the selection of suitable primers, but also comprehensive and sometimes sophisticated bioinformatic analyses for getting meaningful results. This situation puts high demands on the research teams involved and the decision of the method of choice for a specific research question alone can be challenging. One potential option to optimize both time and costs in projects where trophic interactions are to be analysed using DNA‐based approaches is to (partly) outsource this work to the increasing number of specialist commercial providers, as is nowadays routine for Sanger sequencing.

Apart from technical issues, the lack of detailed information on the methodology provided in molecular dietary studies is another challenge for both wet and dry lab procedures. Unfortunately, such reporting standards which are comparable to the minimum information required for qPCR assays in medical applications (*i.e*., MIQE guidelines; Bustin *et al*. 2009). For example, the sensitivity of assays for detecting food DNA is rarely reported and standardized across targets when multiplexing is employed or different diagnostic PCR assays are compared within a study (but see Sint *et al*. 2012). Likewise, assay specificity is often poorly evaluated and documented, for example by sequencing obtained amplicons in diagnostic approaches or detailed reporting of target and nontarget sequence reads in metabarcoding studies. The lack of knowledge regarding such general criteria hampers the comparability of data across studies drastically, providing a significant drawback to utilizing the rapidly growing trophic information generated by DNA techniques.

When it comes to the interpretation of the molecularly generated trophic data several important issues need to be considered. Cannibalistic interactions cannot be depicted as the current approaches allow prey DNA to be differentiated between species but not within species, albeit the first successful efforts distinguishing between fish haplotypes in environmental samples were recently published (Tsuji et al. 2020). Generally, dietary studies still rely on morphological identification and counts of prey remains in parallel to the molecular trophic analyses (*e.g*., Eigaard *et al*., 2014) to assess intraspecific feeding interactions. Furthermore, the molecular approach cannot differentiate the life/developmental stage of the prey and again a combination of molecular and hard part prey remain analysis is advisable in this context, as exemplified by Thalinger *et al*. (2018).

Besides the undisputable advantages and exciting opportunities DNA‐based approaches offer for studying food webs around fish, it should not be forgotten that the combination with other methodologies, such as identification of prey hard parts, stable isotope or fatty acid analysis, can deliver even stronger data sets and ecological insights. For example, Casper *et al*. (2007b), Oehm *et al*. (2017) and Thalinger *et al*. (2018) demonstrate the power of combining prey hard part and DNA‐based analysis. Hardy *et al*. (2010) combined DNA Sanger‐sequencing of fish consumers and clones of prey DNA obtained from stomach samples with microarrays and stable isotope analyses to resolve the carbon flows through food webs in a weir pool on the lower Murray River in southern Australia. In an integrative analysis of *Plectropomus* spp., a fishery targeted species group, in the central Great Barrier Reef, Australia, Matley *et al*. (2018) showed that temporal investigations of dietary patterns *via* molecular prey identification are more accessible in combination with stable isotopes. The diet of the macaroni penguin (*Eudyptes chrysolophus*) was studied using a combination of stable isotope and DNA analysis of faecal samples collected across the different phases of a single breeding season and compared to the penguin diet obtained in a 26‐year dataset of conventional stomach content analysis (Horswill *et al*., 2018). The combined approach offered a less invasive sampling methodology and provided more in‐depth information regarding prey species diversity, making it a promising approach for generating less‐invasive dietary long‐term data sets.
